# Self-efficacy and behavior patterns of learners using a real-time collaboration system developed for group programming

**DOI:** 10.1007/s11412-021-09357-3

**Published:** 2022-01-01

**Authors:** Ting-Chia Hsu, Hal Abelson, Evan Patton, Shih-Chu Chen, Hsuan-Ning Chang

**Affiliations:** 1 Department of Technology Application and Human Resource Development, National Taiwan Normal University, Taipei City, 10610 Taiwan; 2 Department of Electrical Engineering and Computer Science, Massachusetts Institute of Technology, Cambridge, MA 02139 USA

**Keywords:** Block-based programming learning, Online real-time collaboration, Behavior and sequential analysis, Learning analytics, MIT App Inventor

## Abstract

In order to promote the practice of co-creation, a real-time collaboration (RTC) version of the popular block-based programming (BBP) learning environment, MIT App Inventor (MAI), was proposed and implemented. RTC overcomes challenges related to non-collocated group work, thus lowering barriers to cross-region and multi-user collaborative software development. An empirical study probed into the differential impact on self-efficacy and collaborative behavior of learners in the environment depending upon their disciplinary background. The study serves as an example of the use of learning analytics to explore the frequent behavior patterns of adult learners, in this case specifically while performing BBP in MAI integrated with RTC. This study compares behavior patterns that are collaborative or individual that occurred on the platform, and investigates the effects of collaboration on learners working within the RTC depending on whether they were CS-majors or not. We highlight advantages of the new MAI design during multi-user programming in the online RTC based on the connections between the interface design and BBP as illustrated by two significant behavior patterns found in this instructional experiment. First, the multi-user programming in the RTC allowed multiple tasks to happen at the same time, which promoted engagement in joint behavior. For example, one user arranged components in the interface design while another dragged blocks to complete the program. Second, this study confirmed that the Computer Programming Self-Efficacy (CPSE) was similar for individual and multi-user programming overall. The CPSE of the homogeneous CS-major groups engaged in programming within the RTC was higher than that of the homogeneous non-CS-major groups and heterogeneous groups. There was no significant difference between the CPSE of the homogenous non-CS group and the CPSE of the heterogeneous groups, regardless of whether they were engaged in individual programming or collaborative programming within their groups. The results of the study support the value of engaging with MAI collaboratively, especially for CS-majors, and suggest directions for future work in RTC design.

## Introduction

Computer-supported collaborative learning (CSCL) and computational thinking (CT) are regarded as crucial skills today (Stahl et al., 2006). Many successful learning environments were designed specifically for individual learning. It is valuable to consider which of these offer opportunities to introduce meaningful collaborative learning experiences. This study aims to explore a teaching method characterized by the use of a Real-Time Collaboration (RTC) system integrated with a software development environment previously popularized as a platform aimed at supporting students learning individually. The new version of this web-based platform allows many users to collaborate to edit the same software application, including the block-based program and user interfaces, so as to practice CT synchronously and collaboratively. In our current reality, whether it is to develop programming ability or to participate in other large scale projects, opportunities for teamwork are both frequent and indispensable. Therefore, it is not only important for the individual to develop an understanding of CT to enable problem solving, but it is also crucial to do so while having the opportunity to collaborate with others, in part to overcome various challenges that have been noted in the literature (Cen et al., 2016).

Block-based programming (BBP) learning is the method we chose for this study in order to enable participants to solve problems by putting CT processes into practice in realistic situations without requiring proficiency with the syntax of more typical programming languages. In order to reach a broad audience for enhancing CT skills at different teaching stages and for different ages, a review article noted that graphical programming languages or BBP languages are better choices than conventional command-line-based programming languages (Hsu et al., 2018). Learners from all around the world are less likely to be limited by language and syntax when they use BBP to learn CT. A recent study showed that BBP languages were effective for helping students improve their CT ability, and indicated that the effect of MIT App Inventor on basic programming concepts was especially large for students with moderate and low self-efficacy (Tsai, 2019).

Comparing learners with vs. without a teammate, previous research suggested that students in teams enjoyed the problem-solving process more and had more confidence in their solutions (Williams et al., 2001). Therefore, the purpose of the current study was to propose an RTC platform to enhance the MIT App Inventor (MAI), which originally only allowed for individual programming. The aim was to effectively enable versatility in transitioning between collocated and non-collocated collaboration and to increase the flexibility in users’ cooperative problem-solving practices. In sum, the results suggest that RTC allows learners to synchronously and collaboratively complete a programming project with BBP. At the same time, depending on the annotation function and chat room provided by the platform, the analysis reveals how learners communicate with and understand each other’s synchronous behaviors in different ways while communicating the meaning behind the programs in the environment. It is important to note that providing the opportunity to pairs to collaborate within a platform does not guarantee that users will in fact engage collaboratively. And thus it is important in this study to investigate the extent to which collaborative behaviors did or did not occur in pairs and how the presence or absence of such behaviors were associated with outcomes.

In addition to collaboration, which is expected to be a substantial contributor towards learning programming, previous research has indicated that college engineering freshmen gain higher scores on computer skills when they presented higher self-efficacy scores, showing that the correlation between computer skills and self-efficacy scores was statistically significant (Askar & Davenport, 2009). Computer Programming Self-Efficacy (CPSE) is a notable scale to be taken into consideration in connection with learning programming, and has been widely employed and discussed in the research on learning computer programming (Ramalingam & Wiedenbeck, 1998; Kong et al., 2018; Tsai et al., 2019). For example, one past study surveyed 287 primary school students in grades 4 to 6, and the results recommended that future research provide ample collaboration opportunities for students when they learn CT because the students with a more positive attitude toward collaboration had greater self-efficacy, which was beneficial for their learning of programming (Kong et al., 2018). This past work focused on young learners, whereas the focus of the present study is on adult learners, which may exhibit substantially different needs and behavior patterns. Thus, in order to provide more calibrated recommendations for further modification in the development of cooperative learning of BBP, an empirical study was conducted in higher education. The focus was on analyzing the differences in the behavior patterns of learners when they constructed BBP with RTC, which allowed them to collaborate with their partners during the development process. The research questions of the present study are as follows:

1. What are the behavioral patterns of individuals learning BBP, and do they differ between when they working individually or collaboratively?
2. What are differences in the collaborative behavior patterns of the CS-major and non-CS-major adults when they engage in BBP design activities with RTC?
3. How does the CPSE of the adults compare when learning BBP with an individual or collaborative behavior profile with RTC?

## Literature review

### Computational thinking

Computational Thinking (CT), which originates from concepts and processes derived from computer programming and coding, is considered to be a basic skill in the 21st century (Tsarava et al., 2018). CT is also regarded as a competence related to strength and efficiency in problem solving broadly, which thus has a major impact on personal and social development in the information age. Çakıroğlu and Mumcu (2020) have demonstrated that learners perform BBP as problem-solving towards cultivation of CT, and thus engagement with CT thinking processes is compatible with problem solving in connection with computers.

Computer programming offers a fruitful environment for investigation of research questions germane to the field of CSCL. Building on Vygotsky and other social constructivists, CSCL scholars widely advocate for sharing individual perspectives within groups in order to enhance mutual understanding (Alfin et al., 2019). Bause et al. (2018) showed that groups with collaborative support tools exhibit greater intensity of discussion, more balanced discussions, more mutual understanding indicators, and better decision-making performance. Learning in groups can be seen as an intersubjective psychological process in which inspiration from the minds of individual learners produce cognition at the group level (Stahl et al., 2014). Lin et al. (2018) found that a collaborative simulation platform provided a shared space in which learners were able to integrate their perspectives in order to solve problems. Using discourse analysis and epistemic network models, past research suggests that high-performing learners in groups display systematic CT processes, while the CT processes of low-performing teams exhibit patching, guessing, and checking (Wu et al., 2019). Sun et al. (2020) proposed three indicators of collaborative problem solving (CPS), namely constructing shared knowledge, negotiation/coordination, and maintaining team function. However, the behavioral properties of multi-user programming on the online RTC have not been explored. The particular affordances of multi-user programming environments like the online RTC system allow groups to contribute towards authoring of software simultaneously within the same space. The online RTC system is a helpful tool and valuable starting point for enabling synchronous collaboration towards solving programming problems online. The current study delves more deeply in the details and nuances of RTC. The current study further compares the behavior patterns in the BBP and RTC environment with those discussed in the past literature (Sun et al., 2020).

### Online RTC

An online RTC provides users with a platform to synchronously collaborate on editing. The RTC platform is thus a tool for synchronous collaboration. Previous research has confirmed that online-RTC tools are helpful for supporting team members (Hernández-Sellés et al., 2019). Chang et al. (2017) demonstrated that groups were able to use analytical reasoning strategies to solve problems on the collaborative platform. CPS is a skill that allows users to collaborate to solve problems regardless of whether they use online or offline tools. Scholars have defined the skills of CPS as including behavior, collaboration, problem analysis, solution planning, and expanded collaboration in teams (Polyak et al., 2017). CT also involves a similar process; nevertheless, CT is particularly for cultivating students’ understanding of the thinking process and logic that computers accept, while CPS is a good strategy for students to adopt in computationally-focused activities while learning CT.

Due to the benefits and importance of CPS, it is worth developing CPS activities (Graesser et al., 2018) on the BBP online learning platform for students to collaboratively learn CT together. It has been found that online RTC has unique advantages compared with face-to-face learning environments, allowing learners to freely choose their place of study, and thus increasing the flexibility of learning (Jaya et al., 2020). Learning platforms that offer the opportunity for synchronous communication help learners understand each other’s thinking processes through social interactions at a distance, especially when working together to solve new problems (Al-Samarraie & Saeed, 2018).

However, some factors such as prior knowledge, background, and preferences may lead to cognitive biases and can easily be neglected at the planning phase of group learning activities. Learners with different backgrounds have the potential to offer different kinds of support to the group. Fostering effective group dynamics requires identifying and then providing the set of prerequisites for productive discussion (Le et al., 2017). Assessment of the success of CPS also requires a range of expertise as well as it draws from the fields of learning science, data science, psychometrics, and software engineering (von Davier et al., 2017). Thus, new insights regarding collaborative learning at the frontier of computer programming and computational thinking are needed.

### The effects of CS and non-CS majors on learning programming

The learning and promotion of CT is not only beneficial for software developers or computer science (CS) majors, but is also important for non-CS learners. Scholars have found a connection between CT skill development and the strategies for learning and group interaction observed within collaborative learning groups when CT skills are the target (Gong et al., 2020). When contrasting learning of students from a humanities or non-humanities background, no differences in acquisition of CT skills were identified (Katai, 2020). However, Katai (2020) only compared learners with science-oriented and humanities-oriented backgrounds, and did not specifically target learners who were CS vs. non-CS majors. The results may differ since CT skills are so closely related to the process of computer programming. Therefore, this study specifically compared CS-majors and non-CS-majors using the RTC platform to learn CT.

Early research has suggested that while novices might be more likely to solve problems from first principles, experts may be able to access past solutions in an automated fashion, which saves time and effort, and leads to more effective solutions (Wiedenbeck, 1985). Therefore, contrasting CS and non-CS majors may lead to differences in problem solving paths that might further elucidate such findings. Scholars have questioned whether existing online programming tools and instructional programs are able to provide sufficient support for adult learners seeking to improve their practical skills (Zapata-Rivera et al., 2019). Furthermore, past scholars have similarly noted that it is worth exploring non-CS majors’ efficacy in learning CT (Fronza et al., 2019). Accordingly, it makes sense as a research goal to develop more advanced learning support in this area.

Methods are needed for comparison of different CPS environments, including differences in group composition. To that end, researchers have collected learning process data including observations of behaviors in both environments (Lin et al., 2018). In GSEQ, the researchers were able to observe and record the behaviors of the students in videos or logs. Then, the researchers coded the behaviors recorded in the video or logs, and then analyzed the coded data in order to identify frequent behavioral sequences.

### CPSE

In order to evaluate learners’ CT ability, it is necessary to develop more reliable and convenient tools. This is why the CPSE was developed (Tsai et al., 2019; Durak & Saritepeci, 2018) found that the performance of CT was highly positively correlated with thinking style and attitude towards programming classes. Self-efficacy is closely related to the belief in one’s ability to complete specific work, which has been found to be positively correlated with learning motivation (Hsu & Hu, 2017). Since a previous study confirmed that the correlation between computer skills and CPSE was significant (Askar & Davenport, 2009), providing successful and positive programming experiences may be an important goal for the future of education (Tsai et al., 2019). It is reasonable to expect that good performance will trigger learners’ sense of value and encourage them to want to improve their abilities, which might ultimately affect their self-efficacy (Paris & Paris, 2001). Therefore, the current study analyzed the self-efficacy of individual programming and that of multi-user programming in online RTC. It should be noted that this study does not investigate effects on learning of programming or production of high quality software. CPSE is the main success variable.

## Method

### The MAI RTC platform

MAI provides users with a web-based programming environment for writing smart phone applications by BBP (http://ai2.appinventor.mit.edu/). This experiment adopted the MAI platform as a tool for learning programming, and utilized the built-in RTC proposed by Deng (2017) to support online collaboration. Then, the RTC was further enhanced in this study, making it like a Google Document, which allows users to co-edit their blocks in the program. The RTC was mainly used in the study for co-editing and co-creating components and blocks with the BBP tool named MAI. The users were also able to read the notes taken by their team members on the RTC at the same time. Those important changes to the platform made instant co-creation at a distance feasible in the BBP environment, with the goal of broad impact in the present COVID-19 era, specifically in connection with technology education in K-12.

**Fig. 1:**
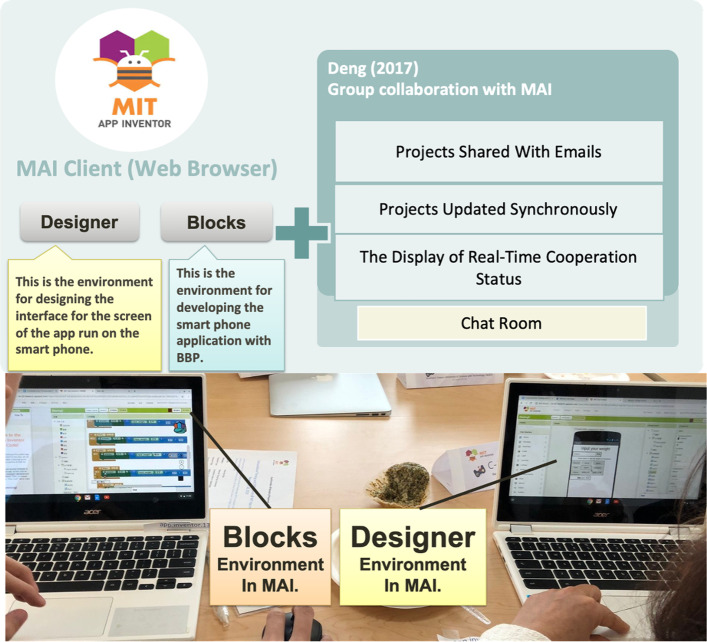
System architecture diagram and a photo of the experiment

The MAI website has two parts, namely Designer and Blocks, which enable users to create their own smart phone applications using the Android system, as shown in Fig. 1. The Designer environment is used for arranging the interface of the application on the screen of the smart phone. The Blocks environment is utilized for writing the block-based programs. The current version has extended Deng’s work by adding a chat room for each group and an annotation function for each block, where users can discuss and interact with their collaborators in real-time to facilitate robust collaboration in a distributed environment.

### Experimental subjects

Because programming experience is expected to lead to different CPSES results (Tsai et al., 2019), this study probed into the participants’ experiences in order to investigate the relationship between processes and outcomes. Participants were considered as part of ability-based clusters, where they were assigned based on the degree of proficiency with using MAI. The participants who were CS majors came in to the study with experience using MAI for more than 3 years. Moreover, they had graduated from CS-related departments, suggesting that they had previously learned other programming languages and had experience with using them for more than 1 year. In this research, a total of 13 participants took part in the experiment (7 males and 6 females), divided into six groups according to their professions.

One group (Group F) was composed of three computer science (CS) background adults familiar with MAI. The remaining groups were each composed of two adults. Group A was composed of two females, both unfamiliar with MAI. Group E comprised two males, both with an education background, and both unfamiliar with MAI. Group D was composed of two males, both familiar with MAI. Group F comprised two males and one female, all familiar with MAI. Group B was composed of two females; one was unfamiliar and the other was familiar with MAI. In Group C, one female was unfamiliar with MAI, and one male was familiar with MAI. The behavior patterns of the different groups’ learning processes were further explored.

Due to the high degree of variation across individuals in the groups and the small sample size, we must abstract up a level in order to draw conclusions while acknowledging potential confounds and limitations on generality. With acknowledgment of the differences in the composition of each group mentioned above, but with reference to the precedent set in a previous study that clustered learners into groups based on the features of their behavioral patterns (Liu & Tsai, 2008; Wen et al., 2018), the participants were labeled either as homogeneous and heterogeneous groups. The homogeneous groups were further grouped into CS majors who were also familiar with MAI, and non-CS majors who were also unfamiliar with MAI, as shown in Table 1.

**Table 1:** Different groupings among the 13 cases

Groups	Clusters	Code of Subject	Major of Subject
A	Homogeneous non-CS group	A-1	non-CS major
		A-2	non-CS major
B	Heterogeneous group	B-1	non-CS major
		B-2	CS major
C	Heterogeneous group	C-1	non-CS major
		C-2	CS major
D	Homogeneous CS group	D-1	CS major
		D-2	CS major
E	Homogeneous non-CS group	E-1	non-CS major
		E-2	non-CS major
F	Homogeneous CS group	F-1	CS major
		F-2	CS major
		F-3	CS major

### Measurement Instruments

To explore the behavior patterns of the users during individual programming and during multi-user programming on the online RTC system, this study adopted behavior sequence analysis with the GSEQ software. The input of the GSEQ includes three parts, the origin of the behavioral codes, the destination of the behavioral codes, and the frequency of the action from one behavioral code to another behavioral code. The setting interface of the GSEQ software is shown in the website (https://www.mangold-international.com/en/products/software/gseq). Firstly, the users had to use the programming website with online RTC when they wrote the programs. Then, the inputs for GSEQ were the logs recorded with a timeline on the programming website. The indicators of actions on the programming website were coded and classified in Table 2, and all behavioral codes were analyzed with the GSEQ 5.1 sequence analysis software. The GSEQ tool was used to calculate the frequencies of one action followed by another, and then to create a transition matrix of learning process actions. The GSEQ software will automatically check all prerequisites and calculate the following formula so as to show the significant frequent behavior sequences on the programming website.

**Table 2:** Schema of behaviors

Categories	Codes		Behavioral explanations
Block		BCH	Revise Blocks
		BCO	Connect to AI companion or Emulator for testing
		BOP	Add a component parameter for a block
		BCC	Add a conditional block
		BMS	Add an arithmetic, logical, or text block
		BMO	Select or move a block
Designer		DCR	Add a component or insert files
		DMO	Select or move a component
		DCP	Set the property of the component
		DEL	Revise/Delete a screen/component
Other		OI	Interact with others by annotation

Note as an example that the Z value of behavior pattern A → B = ((the frequencies of the pattern from behavior A → B) – (the average frequencies of all behavior patterns))/standard deviation, where the symbol of A → B means a sequential behavior pattern in which action A is followed by action B. When the value of z is larger than 1.96, the frequency of the behavioral pattern from one action to another achieves statistical significance.

In addition to the system logs collected above, we also surveyed the users’ study majors and their CPSE. The pre-test included the users’ former experience with programming and their professional major, while the post-test was a survey of their feedback on the course to analyze their viewpoints and feelings when using the RCT platform. Moreover, in order to further improve the present development, this study attempted to understand if there were difficulties in the operating process or aspects of the platform that needed to be improved.

The CPSE scale was employed in this study. This questionnaire uses the 5-point scale developed by Tsai et al. (2019). It includes five dimensions: logical thinking, cooperation, algorithm, control, and debugging. Except for logical thinking, which has four questions, the other dimensions have three each, giving a total of 16 questions. The Cronbach’s α value of overall reliability is 0.96.

### Experimental procedure

**Fig. 2:**
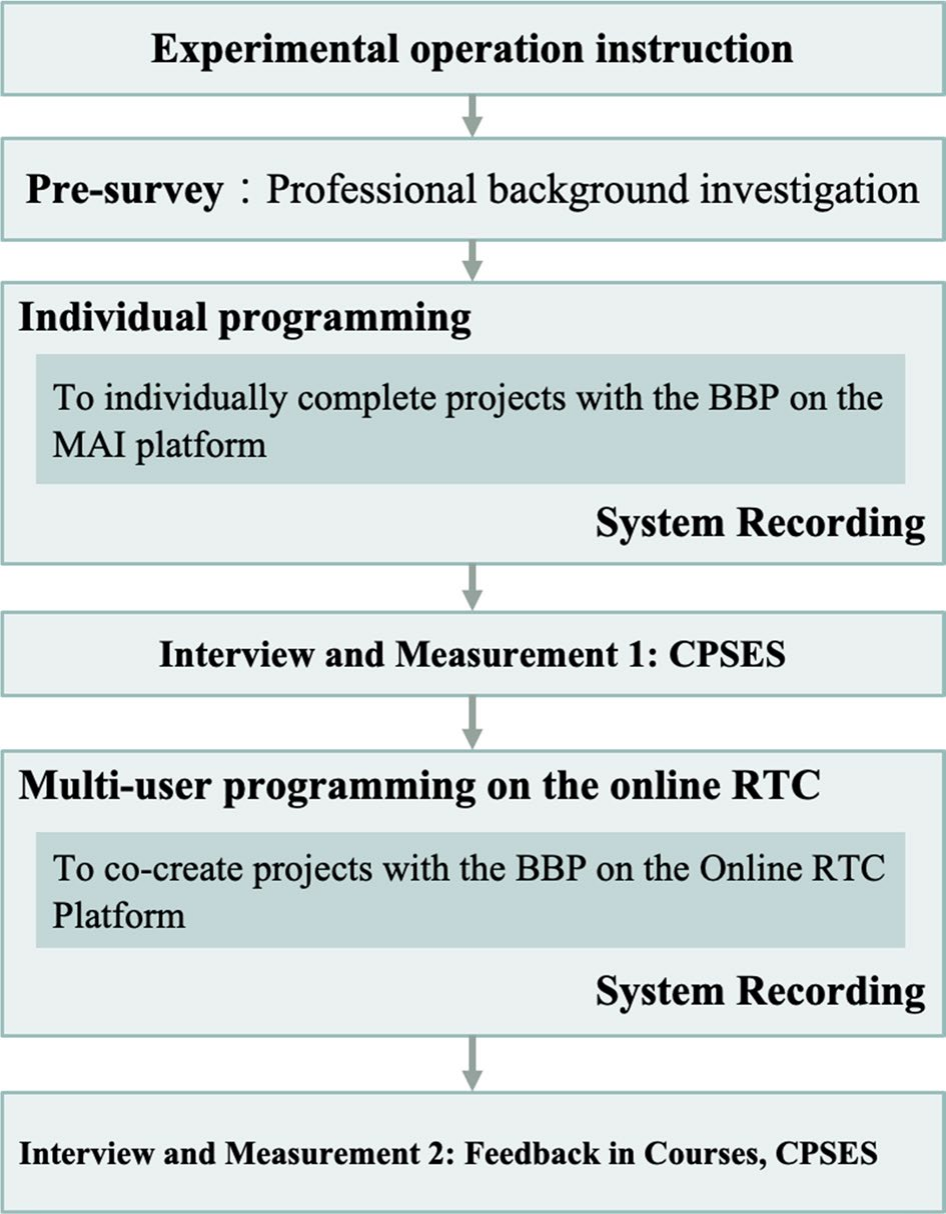
Flowchart of the experimental procedure

All participants were assigned the same tasks in the workshop. This workshop was hosted for half a day in total. This experiment divided the procedure into three major stages: preparation, individual programming, and multi-user programming on the online RTC, as shown in Fig. 2. Firstly, in the preparation stage, the overall experimental process was explained. To understand whether all the subjects had basic knowledge of the MAI platform and operation, we proceeded with basic teaching before the treatment, which included the use of the control components and the parameters in BBP.

Secondly, the users had two projects for individual programming that took approximately 50 min. The first evaluation after the end of individual programming included the evaluation of the CPSE of individual programming and the collection of system operation records (log records) of individual programming during the experiment.

Thirdly, the users had two collaborative projects for multi-user programming on the online RTC, which took approximately 50 min. The second project was an extension of the first project. Therefore, they had to collaborate to solve the first project; otherwise, they could not proceed to the second, extended project. The participants were grouped based on Table 1. This study made sure that groups A to F were balanced based on CPSE because there was no significant difference among the six groups based on the results of the Kruskal Wallis test (*The chi-squared test of groups = 8.241; df = 5; p = 0.143 > 0.05*). The participants had to collaborate to complete the project with RTC. The purpose of developing RTC was to enable the participants to proceed with collaborative learning on the one hand, and to help them coordinate and discuss with others during the project on the other. The teacher played the role of the facilitator only in all stages of the process, and explained the purpose of the app project design before the start of the task. Finally, during the interview and measurement phase, the CPSE scale was investigated, and subjects were asked to give feedback on the operation process with an open-ended question attached in the survey. In addition, the system recorded subjects’ operation process on the platform (RTC). Those logs were used for identifying their significant behavior patterns.

## Results

### The behavioral patterns of individual programming

In this study, the experimental results are explained and presented according to the research questions, which are divided into the behavior mode of individual programming and multi-user programming on the online RTC. We present the results of the experiments in the following sequence: the behavior patterns, the behavior patterns of variously distinguished groups, and the CPSE scale.

In this experiment, the system operation records of 13 cases were collected in the system logs, and their behaviors were coded and processed in chronological order. The obtained data were converted according to the behavior coding shown in Table 2. Finally, a total of 1,897 behavior codes for individual programming were recorded in the logs, as were a total of 3,571 behavior codes for multi-user programming on the online RTC. The transition frequency matrix of the behaviors during individual programming is shown in Table 3, and that of the behaviors during multi-user programming on the online RTC is shown in Table 4. In addition to conducting statistical analyses of the behavior frequency and distribution, each student’s behavior codes were arranged in chronological order to form strings of data before conducting the sequential analysis. The current study then conducted a series of frequency transition matrices to determine the sequential behavioral patterns with GSEQ (Bakeman & Quera, 2011).

**Table 3:** The frequency and distribution of the coded behaviors during individual programming

	BCH	BCO	BOP	BCC	BMS	BMO	DCR	DMO	DCP	DEL	OI	Totals
BCH	9	5	9	3	6	75	2	1	0	0	0	110
BCO	4	4	6	1	0	8	6	6	2	0	1	38
BOP	0	0	0	0	0	77	0	0	0	0	0	77
BCC	0	0	0	0	0	51	0	0	0	0	0	51
BMS	1	0	0	0	0	46	0	0	0	0	0	47
BMO	103	24	56	9	41	545	9	14	5	1	0	807
DCR	0	0	0	0	0	0	0	47	58	0	0	105
DMO	0	0	2	29	0	10	32	253	92	3	1	422
DCP	1	2	3	7	0	5	28	100	89	0	1	236
DEL	1	0	0	0	0	0	0	3	0	0	0	4
OI	0	0	0	0	0	0	0	0	0	0	0	0
Totals	119	35	76	49	47	817	77	424	246	4	3	1897

**Table 4:** The frequency and distribution of the coded behaviors during multi-user programming on the online RTC

	BCH	BCO	BOP	BCC	BMS	BMO	DCR	DMO	DCP	DEL	OI	Totals
BCH	17	2	4	5	10	112	0	5	0	0	1	156
BCO	0	0	0	0	1	4	2	2	0	1	0	10
BOP	0	0	0	0	0	32	0	0	0	0	0	32
BCC	0	0	0	0	0	50	0	0	0	0	0	50
BMS	1	0	0	0	0	48	0	0	0	0	0	49
BMO	136	6	22	33	37	513	4	13	2	0	6	772
DCR	0	0	0	0	0	0	0	44	131	0	0	175
DMO	1	0	4	2	0	5	99	1165	295	38	4	1613
DCP	2	2	2	7	1	7	42	361	219	2	2	647
DEL	0	0	0	0	0	3	15	22	0	0	0	40
OI	0	0	0	1	0	4	4	9	1	0	8	27
Totals	157	10	32	48	49	778	166	1621	648	41	21	3571

In order to further confirm whether there was statistical significance in the behavior comparisons, z-score conversion was performed, as shown in Tables 5 and 6. When the z score is greater than 1.96, these behavioral pattern comparisons achieve statistical significance. That is, the codes shown in the straight line and the codes shown in the column are typical patterns. For example, in Table 5, the BCH→BOP behavior is statistically significant.

**Table 5:** Adjusted residuals table of Z values in the serial behavior analysis of the individuals using BBP

	BCH	BCO	BOP	BCC	BMS	BMO	DCR	DMO	DCP	DEL	OI
BCH	0.85	2.17^*^	2.30^*^	0.10	2.07^*^	5.48^*^	-1.23	-5.56	-4.17	-0.50	-0.43
BCO	1.09	4.02^*^	3.74^*^	0.02	-0.99	-2.77	3.70^*^	-0.98	-1.43	-0.29	3.88^*^
BOP	-2.32	-1.23	-1.83	-1.46	-1.43	10.30^*^	-1.84	-4.81	-3.46	-0.41	-0.36
BCC	-1.87	-0.99	-1.48	-1.18	-1.15	8.32^*^	-1.49	-3.88	-2.79	-0.33	-0.29
BMS	-1.19	-0.95	-1.42	-1.13	-1.11	7.68^*^	-1.43	-3.72	-2.68	-0.32	-0.28
BMO	10.03^*^	3.14^*^	5.60^*^	-3.47	6.28^*^	18.52^*^	-5.59	-18.55	-13.77	-0.71	-1.49
DCR	-2.73	-1.45	-2.15	-1.72	-1.68	-9.17	-2.17	5.67^*^	13.27^*^	-0.48	-0.42
DMO	-6.03	-3.19	-4.20	6.30	-3.71	-19.15	4.16^*^	21.03^*^	6.13^*^	2.54^*^	0.46
DCP	-3.96	-1.22	-2.29	0.40	-2.62	-13.58	6.49^*^	7.89^*^	12.09^*^	-0.75	1.10
DEL	1.55	-0.27	-0.41	-0.33	-0.32	-1.74	-0.41	2.53^*^	-0.77	-0.09	-0.08
OI	0.00	0.00	0.00	0.00	0.00	0.00	0.00	0.00	0.00	0.00	0.00

^*^p < 0.05. BCH: Revise Blocks; BCO: Connect to AI companion or Emulator for testing; BOP: Add a component parameter for a block; BCC: Add a conditional block; BMS: Add an arithmetic, logical, or text block; BMO: Select or move a block; DCR: Add a component or insert files; DMO: Select or move a component; DCP: Set the property of the component; DEL: Revise or remove a screen/component; OI: Interact with others by annotation

**Table 6:** Adjusted residuals table of Z values in the serial behavior analysis of the group using BBP

	BCH	BCO	BOP	BCC	BMS	BMO	DCR	DMO	DCP	DEL	OI
BCH	4.05^*^	2.42^*^	2.26^*^	2.06^*^	5.53^*^	15.47^*^	-2.82	-10.82	-6.01	-1.38	0.09
BCO	-0.68	-0.17	-0.30	-0.37	2.35^*^	1.40	2.31^*^	-1.62	-1.49	2.63^*^	-0.24
BOP	-1.22	-0.30	-0.54	-0.66	-0.67	10.77^*^	-1.25	-5.18	-2.68	-0.61	-0.44
BCC	-1.53	-0.38	-0.68	-0.83	-0.84	13.49^*^	-1.57	-6.49	-3.35	-0.77	-0.55
BMS	-0.81	-0.37	-0.67	-0.82	-0.83	13.01^*^	-1.56	-6.43	-3.32	-0.76	-0.54
BMO	20.24^*^	2.95^*^	6.51^*^	7.99^*^	9.23^*^	33.96^*^	-6.16	-27.55	-14.57	-3.38	0.78
DCR	-2.91	-0.72	-1.29	-1.58	-1.60	-7.16	-3.00	-5.52	19.96^*^	-1.46	-1.04
DMO	-11.47	-2.87	-3.73	-5.75	-6.40	-28.22	3.84^*^	29.23^*^	0.20	6.15^*^	-2.41
DCP	-5.60	0.15	-1.75	-0.64	-2.94	-14.10	2.46^*^	5.87^*^	11.45^*^	-2.21	-1.03
DEL	-1.36	-0.34	-0.60	-0.74	-0.75	-2.20	9.92^*^	1.23	-2.99	-0.69	-0.49
OI	-1.12	-0.28	-0.50	1.07	-0.62	-0.88	2.52^*^	-1.26	-1.95	-0.56	19.81^*^

^*^p<0.05. BCH: Revise Blocks; BCO: Connect to AI companion or Emulator for testing; BOP: Add a component parameter for a block; BCC: Add a conditional block; BMS: Add an arithmetic, logical, or text block; BMO: Select or move a block; DCR: Add a component or insert files; DMO: Select or move a component; DCP: Set the property of the component; DEL: Revise or remove a screen/component; OI: Interact with others by annotation

**Fig. 3:**
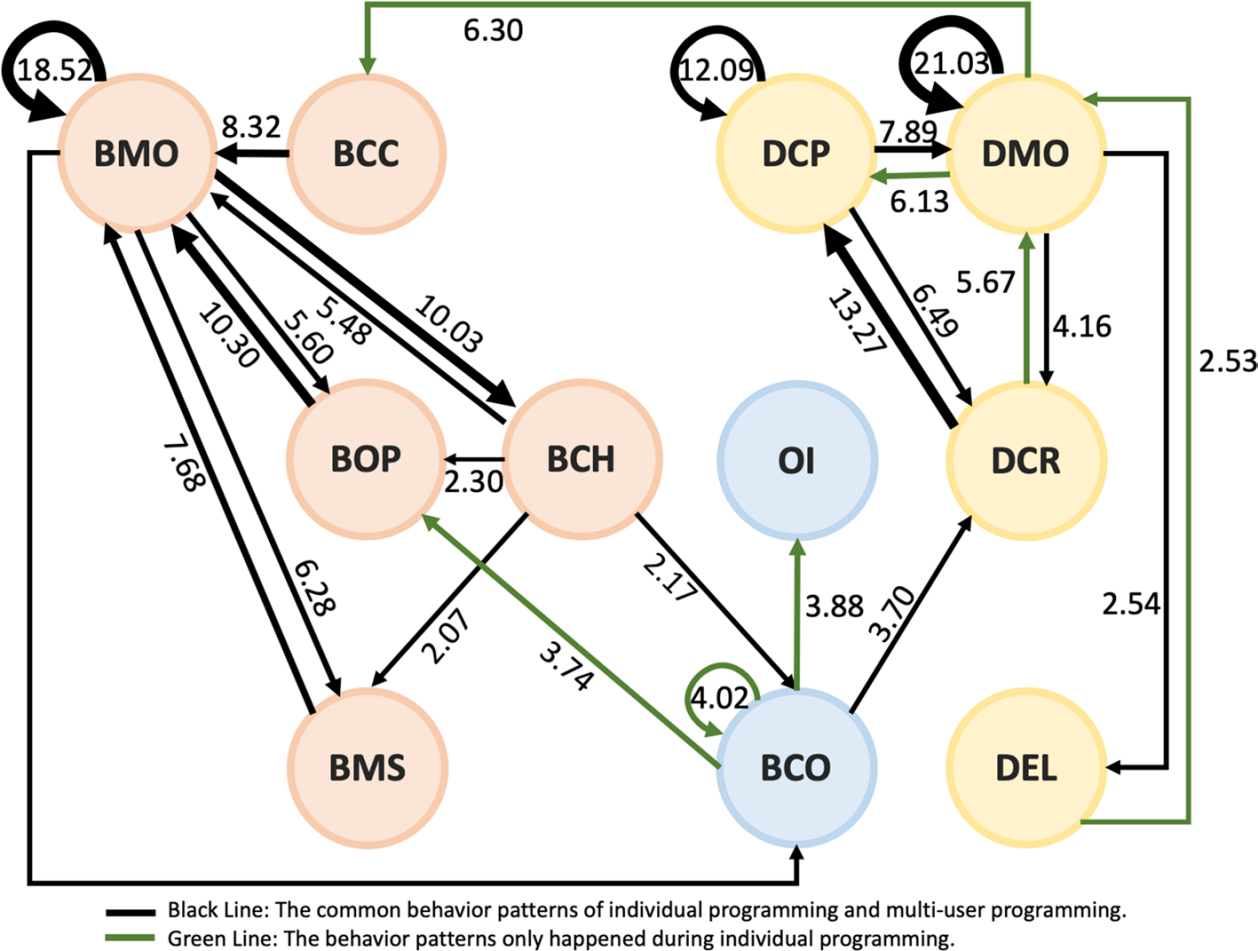
Behavioral patterns of individual programming

**Fig. 4:**
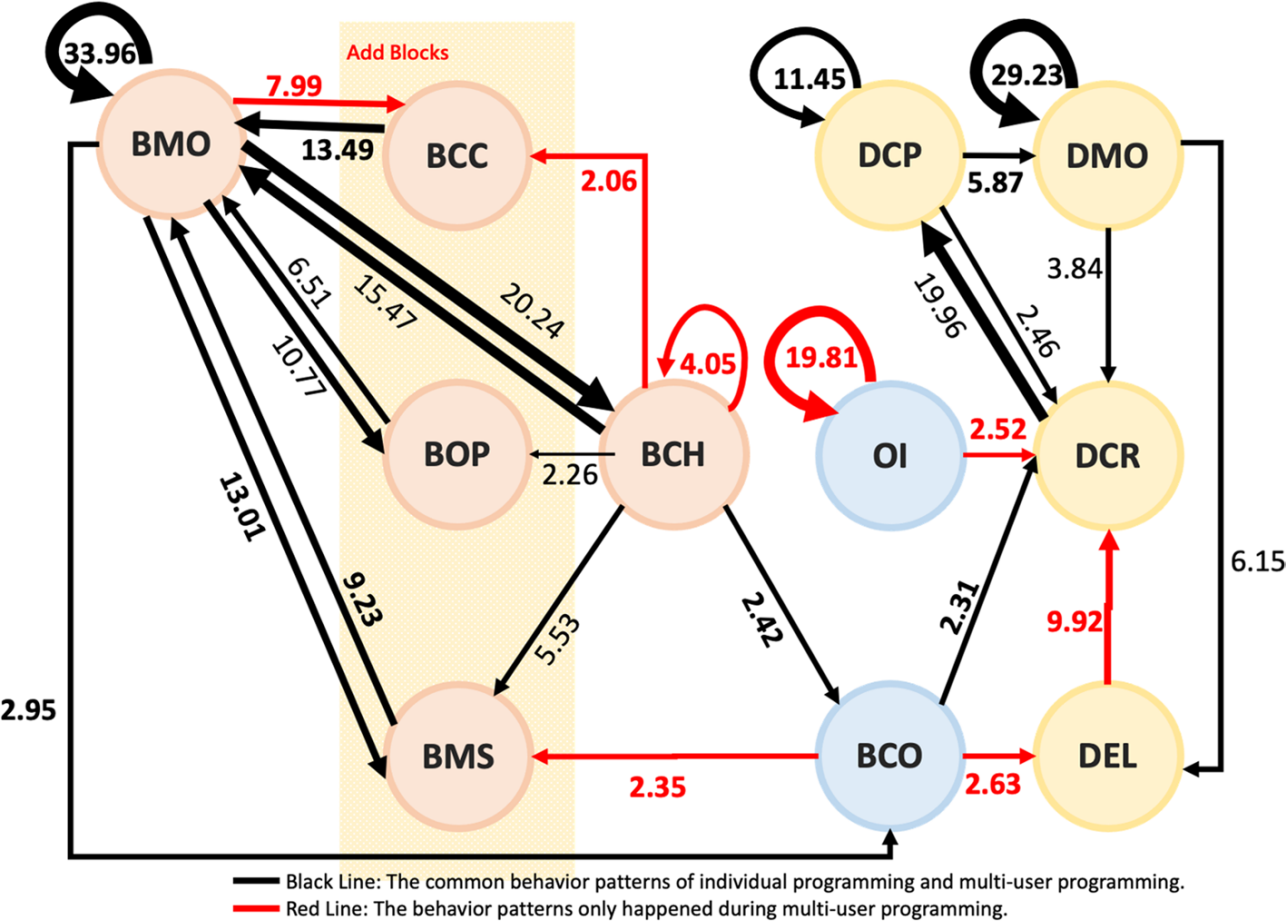
Behavioral patterns of multi-user programming on the online RTC

This study converted statistically significant behaviors into behavior sequential correlation diagrams, as shown in Figs. 3 and 4. In these figures, the black lines represent that both the individual programming and the group-cooperating behavior occur together in the same groups, while the green lines refer to the behavior patterns of individual programming, and the red lines only occur in the behavior patterns of multi-user programming on the online RTC.

Initially, it can be found that in the overall behavior patterns of individual programming and multi-user programming on the online RTC, there was a process of continuous creation, and trial and error correction.

The core of trial and error of interface design for the smart phone application is the behvioral code DEL. The core of trial and error of BBP is the behavioral code BMO. In Fig. 3, it was found that the cycle of trial and error for individual programming with blocks is shown as follows.


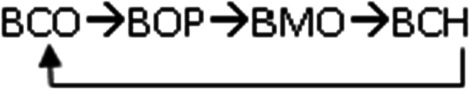


The cycle of trial and error for the interface design of individual programming is DELΔDMOΔDCP, meaning that the user deleted the previous component and went back to re-build a new component and set its properties.

In Fig. 4, RTC achieved the two-way loop, BMOΔBCC, while individual programming only from BMO to BCC. There were more conditions and revisions taken into consideration during multi-user programming on the RTC. In addition, the action of interface design during multi-user programming on the RTC resulted in the significant behavior pattern, DEL◊DCR, so that the cycle in the following was formed.


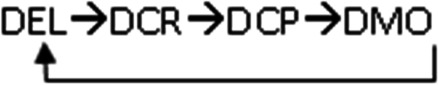


When there was no need to use the RTC functions, the users tended to adopt the simulator repeatedly to confirm the app presentation (BCO) during individual programming, and there was no certain operational sequence in the MAI designer. When the users carried out the multi-user programming on the online RTC, there was more significant communication in terms of interaction (OI), resulting in the following action (DCR).

### Behavior patterns indicative of different clusters

We clustered the groups based on the indicator of whether they were familiar with MAI or not, and analyzed the behavior patterns of each cluster when using the RTC platform with the method of behavior sequential analysis. The clusters are defined as three categories: homogeneous non-CS major, homogeneous CS major, and heterogeneous groups.

#### Cluster 1: Homogeneous group of two non-CS major adults unfamiliar with MAI

There are two groups belonging to the homogeneous non-CS major: group A and group E. According to the system operation recorded files (Logs), there are 240 behavior codes for group A and 325 for group E.

**Fig. 5:**
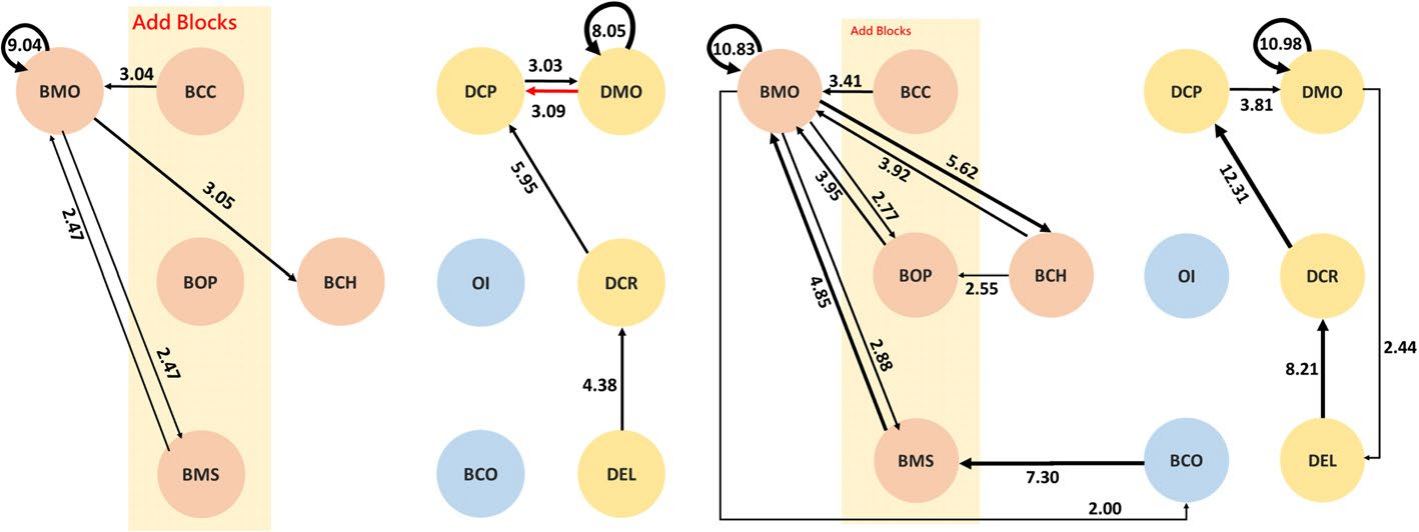
The behavior patterns of two homogeneous non-CS major groups: Group A (left) and Group E (right)

After the behavior coding data were analyzed using the GSEQ software, the converted behavior sequential analysis diagram is displayed in Fig. 5. The black lines indicate that the sequential diagram of the behavior patterns during multi-user programming on the online RTC are significant; the red lines indicate the particular behavior that was only performed by this group.

In the above two behavior sequential analysis diagrams, we can see that Groups A and E have fewer distinguishing behaviors than all subjects, as shown in Fig. 4, and tend to do group interaction (OI) through the platform. Moreover, Group A has even fewer distinctive behavior patterns. The reason may be that this group came into the activity with fewer existing ideas about creation of programs and apps, and so experienced lower group innovation or attempted operations.

#### Cluster 2: Homogeneous group of two CS major adults familiar with MAI

**Fig. 6:**
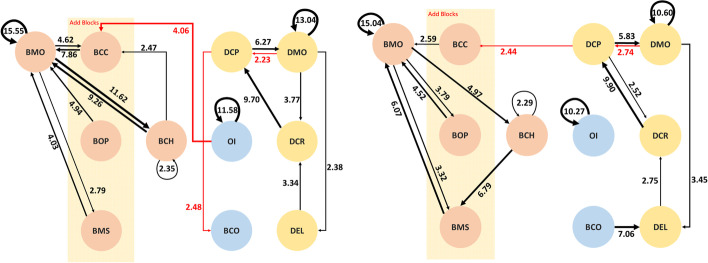
The behavior patterns of two homogeneous CS major groups: Group D (left) and Group F (right)

Groups D and F are in the homogeneous CS major group, for which there are 812 behavior codes for Group D and 507 for Group F. The behavior sequential analysis diagram is displayed in Fig. 6. The black lines indicate that the sequential diagram of the behavior patterns during multi-user programming on the online RTC is significant; the red lines indicate the particular behavior pattern that was specific to this group.

The behavior sequence carried out by the groups of the same CS major are more significant and abundant. Furthermore, there are more diversified behaviors compared with the behaviors of the other groups such as OI→BCC of Group D, DCP→BCO of Group D, and DCP of Group F. The reason for the preliminary estimate is that the subjects of this group were more familiar with information system platforms and program development, and there may have been more complicated cooperation models or role assignments.

#### Cluster 3: Heterogeneous grouping

**Fig. 7:**
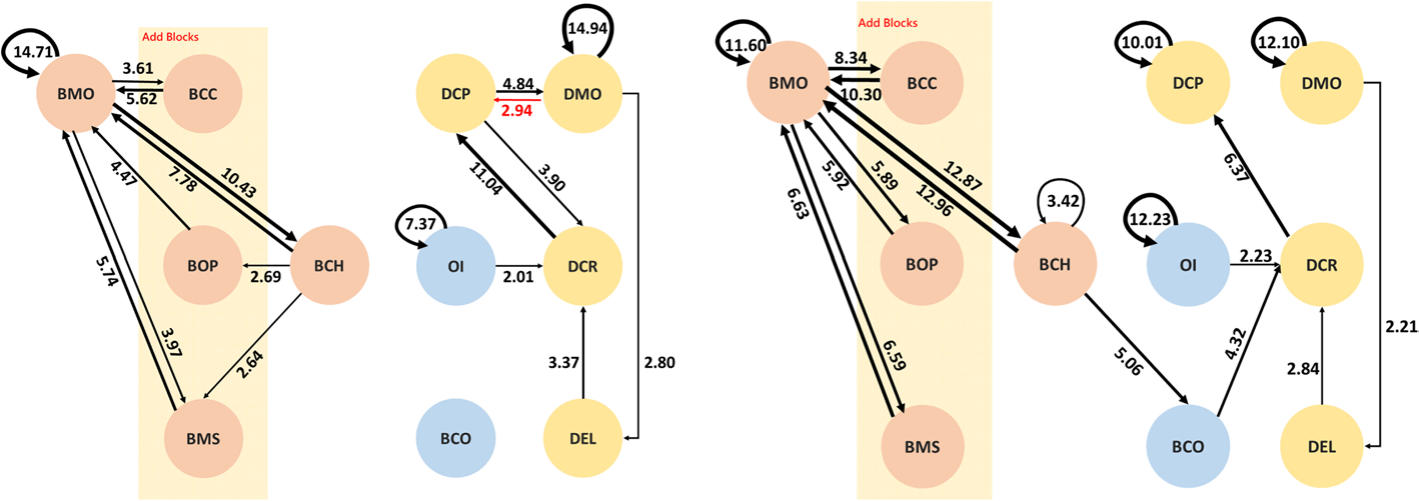
The behavior patterns of two heterogeneous groups: Group B (left) and Group C (right)

There were two heterogeneous groups, Groups B and C, of which one was familiar with MAI and the other was not. There are 782 behavior codes for Group B and 905 for Group C. The behavior sequential analysis diagram is displayed in Fig. 7. The black lines indicate that the sequential diagram of the group cooperative behavior is significant; the red lines indicate the special behavior that was only performed by this group.

Initially, it can be found that the complexity of behaviors of the homogeneous CS majors and non-CS majors in Groups B and C were significantly different. In addition to the basic operation behaviors, the behavior sequential diagrams of the two groups are not consistent. It can be found that Group B has no correlated behaviors between the building block program and the interface design. We conjecture that the factors that resulted in the difference between the two groups are the different uses of cooperation strategies, such as the tendency to work separately, and the tendency to complete part of the synchronization and then to work on it step by step.

### The CPSE performance

The CPSE of individual programming, and that of multi-user programming on the online RTC were investigated. The sample mean CPSE for the three types of grouping are given in Table 7. The three types of grouping were balanced based on CPSE because there was no significant difference among the various groups based on the results of the Kruskal Wallis Test (*The chi-squared test of groups =3.544; p=0.170>0.05*). After the users collaborated to program with RTC, the results indicated that the population mean CPSE was significantly different for the three types of grouping in collaborative learning based on the Kruskal-Wallis Test (The chi-squared test of groups =6.380^*^; *p=0. 041<0.05*).

**Table 7:** Results of the Kruskal-Wallis Test for CPSE of different types of grouping

Treatments	Clusters	*N*	Mean rank	Mean	*SD*	The chi-squared	*df*	*p*
Individual programming	Homogeneous non-CS group (a)	4	6.00	4.27	0.48	3.544	2	0.170
	Heterogeneous group (b)	4	4.88	3.97	0.73			
	Homogeneous CS group (c)	5	9.50	4.71	0.31			
Collaborative learning	Homogeneous non-CS group (a)	4	6.25	4.35	0.52	6.380^*^ (c>a) (c>b)	2	0.041
	Heterogeneous group (b)	4	3.75	3.93	0.57			
	Homogeneous CS group (c)	5	10.20	4.81	0.21			

*p<0.05

In addition, when faced with the characteristics of the various groups, it can be found that the methods of collaboration adopted by the various groups differed in their collaborative processes. This evidence of Table 7 showed that the CPSE of the homogeneous CS-major groups outperformed the CPSE of the homogeneous non-CS-major groups and heterogeneous groups when they collaborated to program with RTC. There is no significant difference between the CPSE of the homogenous non-CS group and CPSE of the heterogeneous group, regardless of individual programming or collaborative programming. In terms of CPSE performance, the overall average of the 13 cases did improve, but did not achieve statistical significance. The phenomenon of the above experiment is discussed in depth in the next section.

## Discussion

### Behavior patterns of individual programming and group operation

During individual programming, the behavior of annotation (OI) was only used for themselves to read later so there was no significant action after annotation. However, during multi-user programming on the online RTC, OI can be used for interacting with teammates through the annotations. It was found that the teammate would come back to Designer to add the component (DCR) after reading the annotations (OI). Therefore, instantly sharing annotations or comments can be regarded as an important function in RTC.

Sun et al. (2020) defined the first indicator of CPS as constructing shared knowledge, while the current study could use OI, which is the behavior of writing annotations in the programs to actively disseminate ideas on the program co-created by a team.

If they did not use OI to share understanding, interruptions would easily occur during multi-user programming on the online RTC. The next step recommended by the current study is to add a new RTC function of approval. When one partner revises any part of the program, regardless of whether it is designer or blocks, the other partner has to decide to approve it or not. If the revision is approved, the present program will be changed. The current study found that the CS-major member of the heterogenous group was confronted with several interruptions from their non-CS-major partner, although the CS-major member was able to share understanding with the non-CS-major member and the non-CS-major member was able to acknowledge the expertise of the CS-major member. The recommended function of approval would solve the negative impact of interruption during BBP because seeking confirmation from other group members about current understanding is effective for collaboration (Jordan & McDaniel, 2014).

From the course feedback after the experiment, it was found that the users had a positive attitude towards RTC. For example, subject D2 commented that, “I can see the execution results at the same time,“ and subject F1 said, “I can observe and we can learn from each other and help each other.“

In the process of project-building and debugging, the users who implemented the program individually adopted the simulator, which is the original function of MAI, to repeatedly confirm their designer and blocks for solving the individual project. When carrying out multi-user programming on the online RTC, the users paid attention to the communication with their partners (OI) and then adjusted directly to the places that needed to be corrected. From the project they worked on, it was clear that the users made great improvements in their conditional logic and interface design, which corresponds to other computational thinking studies (Grover & Pea, 2013; Hsu et al., 2018). Through the RTC platform, the users could not only learn computational thinking with their peers, but could also effectively enhance the opportunities for mutual communication and co-creation.

### Behavior patterns of different grouping clusters

The groups generally set the property of the component first and then moved it to an appropriate place on the screen of the mobile device (DCP→DMO). The special behavior pattern of homogeneous CS-major groups was to adjust the property of the component in the interface design after moving the blocks (DMO→DCP). This is not only a setting behavior but also an adjustment action for ensuring the correctness of the attributes of the component after it was moved to another place. Then, one of the group members returned to write the programs with BBP (DCP→BCC in Group F and DCP→ BCO in Group D).

Oeda and Kosaky (2018) noted that beginners or novices in programming cannot easily comprehend advanced programs written by expert programmers, so they proposed a code-review by using check sheets that enable effective learning between different programmers. With the assistance of the instant changes and annotations in the RTC, the learners were able to instantly do code review in the same group. The RTC provided an instant collaboration platform for different learners in the group. In addition, some significantly different and meaningful collaboration or behavioral patterns were found for collaborative programming with BBP in the current study.

As shown in Figs. 5, 6, and 7, the second research question is answered. Teams with different professional backgrounds have a diversity of meaningful behavior patterns. Based on the results of the time-series behavior analysis and the status evaluation of the course feedback, it was found that the two groups of homogeneous non-CS subjects (Group A and Group E) tended to be split into two significant patterns. One was aiming at the front-end interface design and programming with RTC, and the other was using RTC for instant review and communication with his or her peer while dragging the blocks. They had the behavioral pattern of interacting with others by annotation, which is one kind of peer interaction and communication on the programming platform (OI). A recent study also suggested that a possible future application was to provide hints to novices who frequently reach impasses and are confused by the programming (Jiang et al., 2020). As a result, from the interaction between peers, the novices also receive some new ideas to help them go further (Vygotsky, 1978).

Groups D and F have more significant time-series sequential behavioral patterns, implying that the users with homogeneous CS majors seemed to understand each other’s actions earlier and more easily. It can be speculated that Groups D and F were more familiar with program development using MAI, which led to more complicated cooperation models.

However, in the heterogeneous composition of Groups B and C, the behavior patterns of platform interaction (OI) and simulator use (BCO) are inconsistent. It can be found that the two groups adopted different cooperation strategies. On one hand, it can be found that the homogeneous group tended to split the topic into the two parts of interface and programming when they worked cooperatively on the project with BBP. In addition, they were the first to adopt the cooperative learning mode of dividing the parts to complete them, and to communicate and help each other only when needed. On the other hand, the heterogeneous Group B used collaborative learning, and wrote the code after completing the front-end interface design together, similar to the collaborative learning in previous studies (Dillenbourg, 1999; Roschelle & Teasley, 1995). In addition, it was also found that Group F with the homogeneous CS majors included an additional role of learning leader, which can effectively analyze and discuss according to the status of the topic in real-time, and Group F had a higher degree of participation, which conformed to the contributions of a learning leader (Kim et al., 2020).

Considering the above results, it was found that the RTC platform design does provide some effective RTC to users with different specialties. For example, they can effectively understand each other’s current working status through the prompts of the editing position of the group members. The co-editing programming environment was useful because the team members were able to help each other in a timely fashion, and communicated through the chat room function to achieve the ability to solve collaborative problems of the three aspects of constructing shared knowledge, negotiation/coordination, and maintaining team function (Sun et al., 2020).

### The CPSE performance

BBPs, such as MAI and Scratch, allow learners to write programs with visual blocks that provide useful scaffolding and reduce the cognitive load for novices (Bau et al., 2017). Therefore, young users are able to easily complete a program individually with high CPSE in the BBP environment (Hsu & Hu, 2017). This study further confirms that the users who collaborated to complete a program in the BBP environment enhanced with RTC perceived themselves as having just as high CPSE as those who individually completed a program in the conventional BBP environment without RTC. The overall average was higher than the median score. The mean of the debugging scale self-reported after the individual programming is slightly higher than the mean of the debugging scale self-reported after multi-user programming on the online RTC, although the difference did not reach statistical significance. From the interviews, this study inferred that the RTC may not fully prevent the interruption of the collaborators because the users were forced to accept another collaborator’s revision on the blocks or components by default. Therefore, in the future, it is suggested that RTC should provide one more confirmation button, which will allow the users to choose whether to accept their collaborator’s change in the co-editing program.

In general, the mean of CPSE, regardless of individual programming or multi-user programming on the online RTC, was higher than 3.5, which is the threshold on a 5-point Likert scale. After using the RTC platform, the subjects still retained their belief in achieving a high degree of success in performing programming tasks. The average percentage of being cooperation-oriented was as high as 90%, which shows that the RTC is capable of assisting the development of cooperation in programming, in particular for those who are CS-majors.

## Conclusions

This research argues that the area of adult collaborative learning of computer programming is an area needing further attention and specifically contributes to the development of a specific collaborative platform for BBP. The operational behavior patterns of the RTC platform were collected and analyzed in this study, and the cooperative behaviors of learners with different professional backgrounds were identified, thus revealing notable differences that should be investigated further in future research. The research results show that there are more trials and debugging processes when working with RTC platforms, and adjustments are made through the real-time nature of interaction on the platform to achieve co-creation. Observing this process reveals some of the inner-workings of through during computer programming that are invisible during individual programming, and less visible when collaboration occurs asynchronously, for example in opensource software communities. Furthermore, group members with different backgrounds also display different strategies and behaviors that might be instructive when considering collaboration platforms or related courses, especially for adult learners with diverse backgrounds. The RTC platform proposed in this study allows multiple tasks to happen simultaneously, which affords a high degree of contingent behavior. For example, we frequently observed a user arranging components in the interface design while another user dragged blocks to complete the program in a different window.

The current study is indicative of new questions and insights, and yet we must acknowledge the limitations in terms of total classroom time and the number of participants, and thus we call for future research to build on and extent these findings. For example, it is an open question whether the age of users has an impact on effective use of the RTC system, which raises questions about the appropriate timing for users to adopt the RTC platform during their learning trajectory. This study developed and evaluated the RTC in an actual classroom so that all the participants were able to be observed and video-recorded. In the future, it would also be valuable to conduct studies of online collaborative learning of users across countries. This research verifies that the RTC platform effectively helped users from a different culture to cooperate with one another. And thus we highlight the opportunity to expand on these findings with additional work in the area of cross-cultural CSCL.

This study is supported in part by the Ministry of Science and Technology under contract numbers MOST 108-2511-H-003 -056 -MY3 in Taiwan, and the RTC platform is supported in part by the project supervised by Hal Abelson granted from the Hong Kong Jockey Club Charities Trust.

## Data Availability

The data used to support the findings of this study are available from the corresponding author upon request.

## Code availability

The data used to support the findings of this study are available from the corresponding author upon request.
